# Supplementary figures and images for: Genome-wide association studies reveal potential candidate genes associated with amino acid in tea plants
Source: PeerJ. 2025 Mar 4;13:e18969. doi: 10.7717/peerj.18969 (PMC11887564; doi:10.7717/peerj.18969)

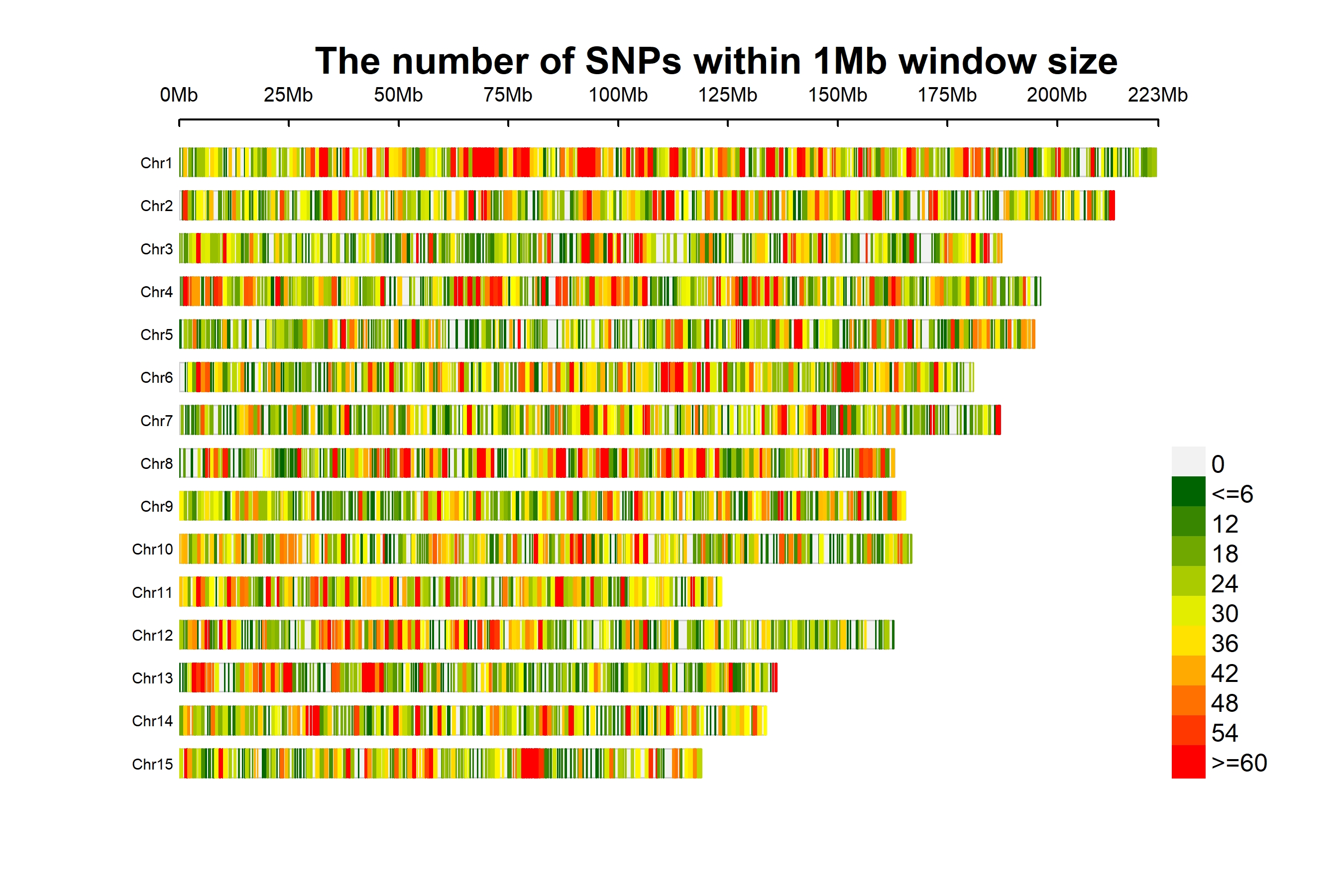

Supplement: Figure S1 — The horizontal axis shows the chromosome length. [file peerj-13-18969-s001.jpg]

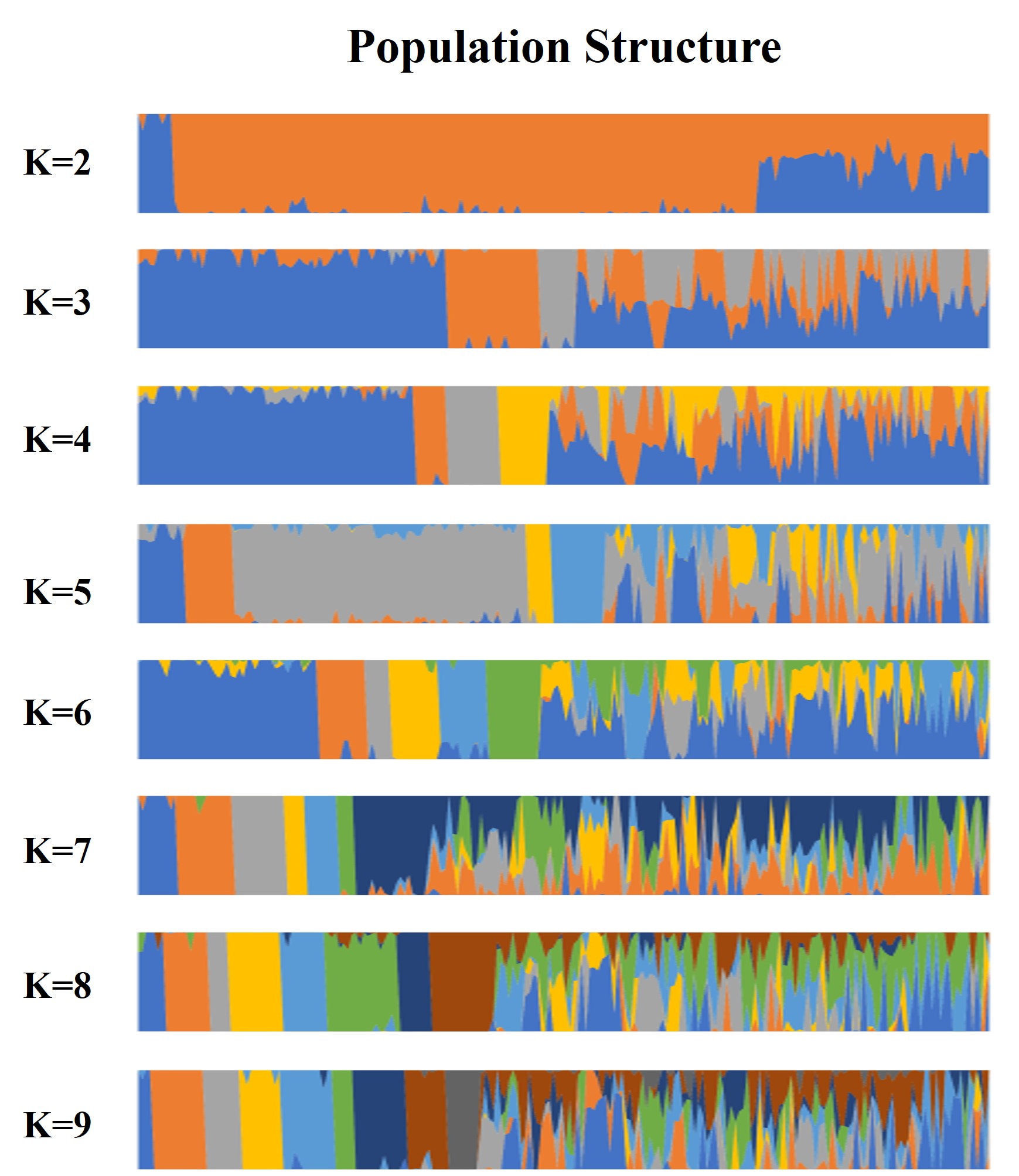

Supplement: Figure S2 [file peerj-13-18969-s002.jpg]

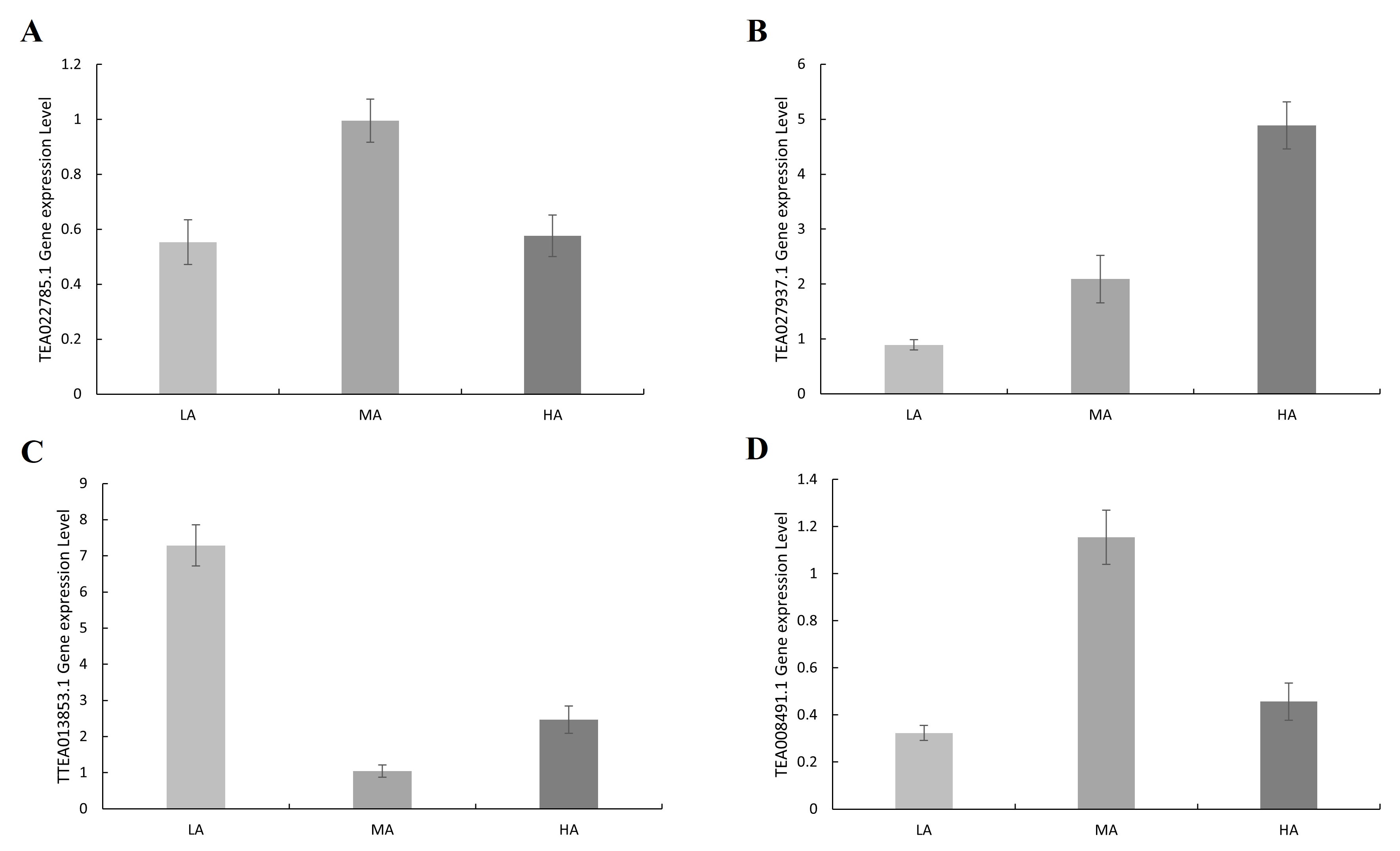

Supplement: Figure S3 — HA, leaves with high amino acid content; MA, leaves with moderate amino acid content; LA, Leaves with low amino acid content. [file peerj-13-18969-s003.jpg]
